# Investigating the Antimicrobial Efficacy of Cannabinoids and Their Derivatives Against *Neisseria Gonorrhoeae* by Computational Analysis

**DOI:** 10.3390/biology14091272

**Published:** 2025-09-15

**Authors:** Umairah Natasya Mohd Omeershffudin, Zakirah Zainal Abidin, Zaw Myo Hein, Che Mohd Nasril Che Mohd Nassir, Ebrahim Nangarath Kottakal Cheriya, Suresh Kumar, Muhammad Danial Che Ramli

**Affiliations:** 1 Schools of Graduate Studies, Post Graduate Centre, Management and Science University, University Drive, Off Persiaran Olahraga, Section 13, Shah Alam 40100, Selangor, Malaysia; umairahnatasya@yahoo.com (U.N.M.O.); zakirahzaenalabideen@gmail.com (Z.Z.A.); 2 Department of Basic Medical Sciences, College of Medicine, Ajman University, Ajman P.O. Box 346, United Arab Emirates; z.hein@ajman.ac.ae; 3 Department of Anatomy and Physiology, School of Basic Medical Sciences, Faculty of Medicine, University Sultan Zainal Abidin, Kuala Terengganu 20400, Terengganu, Malaysia; nasrilnassir@unisza.edu.my; 4 Department of Physiology, International Medical School, Management and Science University, Seksyen 13, Shah Alam 40100, Selangor, Malaysia; ebrahim_nangarath@msu.edu.my; 5 Faculty of Health and Life Sciences, Management and Science University, Seksyen 13, Shah Alam 40100, Selangor, Malaysia

**Keywords:** molecular docking, *Cannabis sativa* L., antibiotic, cannabinolic acid, iron sulfur cluster

## Abstract

*Neisseria gonorrhoeae* is a Gram-negative diplococcus that causes gonorrhea through sexual contact. This ancient STD remains a major public health concern due to reproductive health impacts, antimicrobial resistance (AMR), and lack of a vaccine. Cannabis sativa contains antibacterial cannabinoids, though its role in combating antibiotic resistance is underexplored. The 2Fe-2S iron–sulfur cluster protein is a potential antibiotic target, as these clusters are vital for bacterial proteins involved in electron transport, enzyme activity, and gene regulation. Disrupting them may impair bacterial survival and function. In this investigation, the 2Fe–2S iron sulfur cluster binding domain-containing protein (NGFG_RS03485), identified as a potential therapeutic target from the core proteome of 12 *Neisseria gonorrhoeae* strains, was selected for this study. Potential antimicrobial agents were explored through molecular docking studies involving 16 cannabinoid analogs—9 obtained from literature sources and 7 identified via fingerprint similarity searches. The study revealed that four cannabinoids form favorable bonds with active regions against our targeted protein; with a high binding affinity formed from the molecular docking; 1,3-Benzenediol, 2-[3-methyl-6-(1-methylethenyl)-2-cyclohexen-1-yl]-5-pentyl-, (1R-trans). Dronabinol, Cannabinolic acid A (CBNA), Cannabigerolic acid (CBGA), and Ferruginene C are derivatives identified. Drug-likeness assessments were conducted to evaluate the pharmacokinetic and toxicity properties of the cannabinoids and compared against the antibiotics.

## Simple Summary

*Neisseria gonorrhoeae*, the bacterium responsible for gonorrhoea, has developed increasing resistance to multiple antibiotics, making new treatment strategies urgently needed. This study explores the potential of cannabinoids and their derivatives as antimicrobial agents targeting *N. gonorrhoeae*. Using computational methods, including molecular docking and fingerprint-based compound searches, the study identified five promising cannabinoid compounds with strong binding affinities to the 2Fe-2S iron–sulfur cluster binding domain-containing protein, a critical bacterial enzyme involved in electron transport and cellular function. These include 1,3-Benzenediol (a cannabidiol derivative), Ferruginene C, Dronabinol, Cannabinolic acid A (CBNA), and Cannabigerolic acid (CBGA). Their interactions were visualized using PyMOL and PLIP, revealing significant hydrogen bonding and hydrophobic interactions at active binding sites. Additionally, drug-likeness and pharmacokinetic assessments were performed, showing favorable absorption and low toxicity for several compounds compared to standard antibiotics. Importantly, these cannabinoids showed potential to disrupt bacterial metabolic processes without inducing typical resistance pathways. The findings support further exploration of Phyto cannabinoids as natural alternatives for treating multidrug-resistant N. gonorrhoeae, with the 2Fe-2S cluster protein as a novel target. Further in vivo validation is recommended to confirm their therapeutic efficacy and safety.

## 1. Introduction

*Neisseria gonorrhoeae* is a Gram-negative, nonmotile, nonsporulating diplococci that is predominantly aerobic; however, it can proliferate in microaerophilic environments. The bacteria are also known as bacterial sexually transmitted infections (BSTIs). Since the nineteenth century, sexually transmitted illnesses caused by this bacterium have been recognized as a public health hazard. Resistance to penicillin, fluoroquinolones, sulfonamides, tetracycline, macrolides, and, more recently, extended-spectrum cephalosporins (ESCs) (cefixime and ceftriaxone) and azithromycin has gained prominence in recent decades resulting in significant morbidity and economic cost on a global scale [1,2,3,4]. In 2019, Centers for Disease Control and Prevention (CDC) classified *Neisseria gonorrhoeae* as one of the “nightmare bacteria” in the post-antibiotic era [5].

According to the World Health Organization (WHO), it is estimated that approximately 82.4 million reported cases of gonorrhea majority in occurred in the WHO’s African and Western Pacific regions. As reported by Farha (2020) and Paritala (2013), there were approximately (47.7 million–130.4 million) adolescents and adults aged 15 to 49 years, with a worldwide incidence rate of 19 [6,7] per 1000 women and 23 [8,9] per 1000 males [10]. While research has produced a considerable number of new antibiotics over the previous century, the number of antimicrobial agents discovered has been declining since the 1990s, contemporary with an alarming growth in the phenomena of antibiotic resistance [11]. The surging phenomena of antimicrobial resistance suggest an urgency to identify a new antimicrobial agent and alternative therapies to threaten multidrug-resistant pathogens. *Cannabis sativa* L. biosynthesizes an arsenal of resorcinyl core decorated scaffolds with para-oriented terpenyl, pentyl, and isoprenyl groups, which are known as phytocannabinoids and have a wide therapeutic range, including anti-cancer, anti-epileptic, and analgesic properties [12]. While it has been known that this plant contains antibacterial cannabinoids, their potential to combat antibiotic resistance has received just a cursory examination [11].

Cannabinoids are structural components found largely in the cannabis plant and many animal beings, as synthesized chemicals, or as chemical compounds created by numerous biological species. Phyto cannabinoids, also known as exogenous cannabinoids, are cannabinoids derived from the cannabis plant’s glandular trichomes. THC and CBD are the most evident cannabinoids. Cannabinoids bind to the CB1 and CB2 cannabinoid receptors in humans [12]. They are found all over the body, including in immune cells [12]. Triggering these receptors could affect the immunological response to infection [8].

Recent discoveries showed that CBD could be used as antimicrobials that target Gram-negative bacteria, especially the “urgent danger” disease *N. gonorrhoeae* [6]. Most importantly, the study showed that repeated use of CBD does not lead to resistance against these pathogenic bacteria [13]. This combination of characteristics presents a compelling argument for pursuing additional research on this understudied class of chemicals. This study focuses on the 2Fe-2S iron–sulfur cluster binding domain-containing protein (NGFG_RS03485), identified as a potential drug target from the core proteome of 12 strains of *Neisseria gonorrhoeae* in our previous research using subtractive genomics approach [14].

Research has shown the critical role of iron–sulfur clusters in bacterial physiology and antibiotic resistance mechanisms. For instance, in Escherichia coli, the Ferric uptake regulator (FUR) binds a (2Fe-2S) cluster to sense intracellular iron levels, while the Feo system, responsible for ferrous iron transport, contains a (4Fe-4S) cluster in its FeoC protein. Additionally, SoxR, a bacterial iron–sulfur regulatory protein, acts as a sensor-switch to help bacteria adapt to various stress conditions, such as oxidative or nitrosative stress. Understanding how the 2Fe-2S iron–sulfur cluster binding domain-containing protein contributes to bacterial physiology and antibiotic resistance mechanisms could lead to the development of new strategies to combat resistant infections [15].

This study aims to identify the most promising phytochemicals and cannabinoid-derived compounds that could serve as potential candidates for the development of novel antibacterial drugs against *Neisseria gonorrhoeae* and other multidrug-resistant pathogens. The primary objective is to conduct molecular docking analyses to evaluate the inhibitory potential of these compounds against the 2Fe-2S iron–sulfur cluster binding domain-containing protein (NGFG_RS03485) of *N. gonorrhoeae*. Additionally, the study will assess the physicochemical properties, pharmacokinetics, and toxicological profiles absorption, distribution, metabolism, and excretion (ADME) of the selected compounds to determine their drug-likeness. The findings were expected to contribute to ongoing efforts in addressing antibiotic resistance and discovering new therapeutic options for bacterial infections. In continuation of previous research, the present study sought to examine the medicinal potential of cannabidiol and other cannabinoid compounds with a high degree of similarity through computational studies to evaluate their inhibitory action on the 2Fe-2s iron–sulfur cluster binding domain-containing protein (NGFG RS03485) of *N. gonorrhoeae*. Iron–sulfur clusters are essential cofactors in many proteins involved in various cellular processes, including electron transport, enzyme catalysis, and gene expression regulation. Targeting proteins with iron–sulfur clusters in bacteria can disrupt crucial cellular functions, making them promising targets for antibiotic development [14].

## 2. Materials and Methods

### 2.1. Binding Site Prediction of the Protein

DoGSiteScorer is a grid-based approach that employs a Gaussian filter to identify possible binding pockets based only on the protein’s three-dimensional (3D) structure. The general attributes such as the chemical composition, size, and shape were predicted including the (sub)pockets. The greater the drug score, the more likely the pocket is to be druggable [15]. The binding site of the identified potential drug target protein; 2Fe-2s iron–sulfur cluster binding domain-containing protein; NGFG RS03485 (NCBI Accession: WP_003688839) of *N. Gonorrhoeae* PDB format identified from previous study was accessed using DoGSiteScorer (accessed on January 2025) [16].

### 2.2. Fingerprint Search

In this study, 9 main known cannabinoids were chosen based on literature findings and utilized in our study as ligands for molecular docking (Table 1) [13]. These cannabinoids are then fingerprinted to identify similar substructures to discover other natural products. The natural product activity and species source (NPASS) database contains experimentally determined activity attributes and sources for 35,032 natural products (NPs) from 25,041 species that target 5863 targets (2946 proteins, 1352 microbial species, and 1227 cell lines) [12]. To identify the similar structure of the ligands, they are screened by using PubChem 881-bit (accessed on 1 January 2025) substructure fingerprint search using NPASS (http://bidd2.nus.edu.sg/NPASS/) (accessed on 1 January 2025) tools with default setting subjected to search against PubChem database with 881-bit substructure and threshold set to ≥99 (99%) similarity to identify the similar compound. The aim of the fingerprint searches was to identify and evaluate compounds that have similar structures to cannabinoids and ultimately to be further explored as antibiotics to treat *N*. *gonorrhoeae*.

### 2.3. Molecular Docking

The ligands of the cannabidiol analogs, identified cannabinoids, and derivatives from the fingerprint search 2D structure (accessed on 1 January 2025) were retrieved from the PubChem server (https://pubchem.ncbi.nlm.nih.gov/) (accessed on January 2025) in SDF format. The 2D structure sdf file was converted to PDB format by using Open Babel [12]. The cannabinoids identified from the fingerprint search were also chosen as the inhibitor ligand.

Autodock Tools (ADT) MGLTools v1.5.7 was used to prepare the receptors and ligands input file. Assigning Gasteiger charges and merging nonpolar hydrogen atoms during the docking process, each torsion was permitted to rotate. Grid maps for the protein are generated using the auxiliary program AutoGrid v4.2.9. Each grid was centered on the receptors of the respective cell type. The grid dimensions used for blind docking targets on the active binding site were identified. Using Autodock Tools, the protein receptor and ligand PDB format was converted to pdbqt. Hydrogen was added to the compound for better optimization, and Gaisteger charges were computed. The torsion root was defined for the ligands. X, Y, and Z grids were computed with 1 A for the protein receptor. For all protein receptors, the exhaustion was set to 8 by default. The docking score was evaluated by the docking energy score (kcal/mol). Ligands with the lowest binding affinity will be considered potential drug target candidates.

### 2.4. Evaluation of Protein-Ligand Interaction

pyMOL was used to perform molecular visualization of the polar and non-polar interactions for the proteins to evaluate the molecular interactions between the ligands and the targeted molecules [17]. The protein–ligand interaction profiler (PLIP) was also utilized to interpret the protein–ligand interactions. PLIP is an open-access web-based tool (https://plip-tool.biotec.tu-dresden.de/plip-web/plip/index; accessed on 1 January 2025) that analyzes and visualizes protein–ligand interactions and provides output data in formats suitable for further processing [18].

### 2.5. Comparative Drug-likeness Assessment of Identified Cannabinoids and Its Derivatives Against Antibiotics Used in Treating N. gonorrhoeae Infection

To further identify the drug-like capability of the identified ligands before performing molecular docking, the ligands were examined based on 5 criteria of the Lipinski rules and orally examined active molecules were investigated to establish physicochemical ranges that have a high possibility of becoming oral drugs. This Rule-of-five (Ro5) outlined the association between pharmacokinetic and physicochemical properties. A drug-like molecule would consist of an orally accessible molecule that meets Lipinski’s rule and demonstrates a balance between lipophilicity and hydrophilicity [19]. Ro5 states that any chemical compound was impermeable or poorly absorbed if it violates two of the following criteria: molecular mass 500 Da, hydrogen bond no greater than five bond donors, less than ten hydrogen bond acceptors, and an octanol–water partition coefficient log P not more than five [20].

The Brain or Intestinal EstimateD permeation method (BOILED-Egg) was used to distinguish drugs by predicting the physicochemical space of molecules with a high probability of penetrating the gastrointestinal tract or permeating the brain [21]. The BOILED-Egg predicts the passive gastrointestinal absorption; Human Intestinal Absorption (HIA) and brain access; Blood–brain barrier (BBB) of small-molecule drugs that are useful for drug discovery and development. The evaluation of the Lipinski rules and BOILED was performed by using SWISSADME (http://www.swissadme.ch/) (accessed on 1 January 2025) [22].

The compound’s pharmacokinetic profile assessment of defined absorption, distribution, metabolism, excretion (ADME), and toxicity properties was analyzed via pkCSM (accessed on 1 January 2025) tools that are publicly available (https://biosig.lab.uq.edu.au/pkcsm/prediction) [23]. The organ-specific toxicity and toxicity end points predictions that includes hepatoxicity (liver), neurotoxicity (nervous system or brain), nephrotoxicity (kidney), mutagenicity (mutations in DNA) were assessed by using ProTox-II (https://tox-new.charite.de/protox_II/, accessed on 1 January 2025) that estimate the likelihood of adverse effect on the associated organs. Hepatoxicity model in ProTox-II were based on the DILI model that was trained against 850 compounds and validated via 10-fold cross-validation (AUC = 0.94) using Random Forest with molecular fingerprints. While for neurotoxicity, the model used was trained on 550 compounds and validated by 10-fold cross-validation (AUC = 0.87) with Random Forest and SMOTE-VDM sampling [22].

Nephrotoxicity was based on a model trained with 811 compounds, validated via 10-fold cross-validation (AUC = 0.86) using Random Forest with kMedoids2 sampling. As for mutagenicity, ProTox-II uses machine learning with strong accuracy (0.84 balanced accuracy). The prediction model was based on molecular fingerprints and validated against Ames Test data.

## 3. Results and Discussion

### 3.1. Ligand Selection and Identification of Natural Products Through Fingerprint Searches

A study conducted by Blaskovich et al. revealed cannabidiol’s outstanding anti-biofilm action, low propensity to cause resistance, and topical in vivo efficacy [13]. The study was the first to report that cannabidiol preferentially kills a subset of Gram-negative bacteria, including the “urgent danger” disease gonorrhoeae (ATCC 19424) and hence nine of the main cannabinoids were selected (Table 1). Our study focused on analyzing the major cannabinoids that have been identified through extensive literature searches. This approach was crucial as it guarantees a comprehensive examination of the most pertinent and extensively researched cannabinoids.

To identify other similar natural products that have similar substructures to the identified cannabinoids, the compound was subjected to fingerprint searches. These searches enable us to thoroughly examine the distinct molecular attributes of the found cannabinoids. The chemical similarity search was conducted using a threshold of ≥0.99 as established by the Tanimoto coefficient cutoff. There has been an upsurge in examining natural products as potential medication candidates and the ongoing investigation of traditional and herbal remedies’ therapeutic claims and processes in recent years.

Based on the fingerprint search analysis, Cannabidivarin and Cannabidiol have similar structures with chemical formulae of C_19_H_26_O_2_ and C_21_H_30_O_2_, respectively. The results are tabulated in Table 2.

### 3.2. Molecular Docking Analysis of Cannabinoids and Its Derivates Against N. gonorrhoeae; 2Fe-2s Iron–Sulfur Cluster Binding Domain-Containing Protein

2Fe-2S, commonly known as ferredoxins, are iron–sulfur proteins (Fe-S) that operate as an electron-mediating catalyst to permit the biological production or utilization of hydrogen gas by bacteria [24]. This cluster protein, which contains two iron atoms and two inorganic sulfur atoms as bridge ligands, plays an essential role in bacterial pathogenesis as the innate virulence factor [25]. Iron–sulfur clusters are critical cofactors for numerous types of proteins and are involved in a variety of cellular activities, such as electron transfer, nitrogen fixation, and gene regulation [26]. Several studies have shown that redox metabolism was viable when designing anti-infectious medications, and iron–sulfur proteins have been specifically implicated as a promising target [27]. In addition, microbial sulfur metabolic pathways are generally lacking in humans, making them exceptional therapeutic intervention targets [7]. Several studies suggest the significant role of the cluster of iron–sulfur clusters in the survival of *Y. pseudotuberculosis* in the spleen, and that these extracellular bacteria depend on this route for survival inside host organs [28].

**Figure 1:**
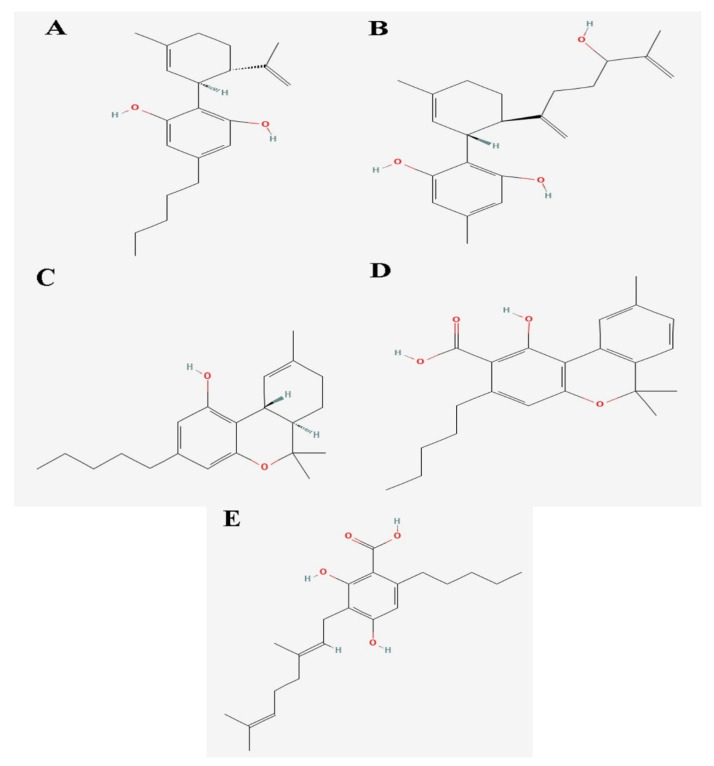
2D structure of identified cannabinoids and derivatives as potential drug candidates of *N. gonorrhoeae* (**A**) 1,3-Benzenediol, 2-[3-methyl-6-(1-methylethenyl)-2-cyclohexen-1-yl]-5-pentyl-, (1R-trans)-. (**B**) Ferruginene C (**C**) Dronabinol (**D**) Cannabinolic acid A (CBNA) (**E**) Cannabigerolic acid (CBGA).

**Figure 2:**
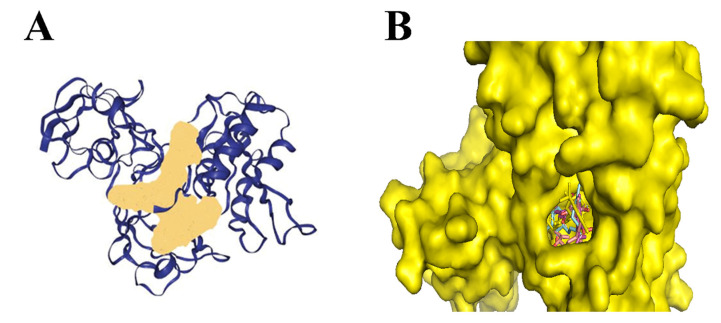
(**A**) The active site is demonstrated by the region on a colored peach is the binding site of on chain A of the protein predicted by DoGSiteScore (**B**) Docking of 1,3-Benzenediol, 2-[3-methyl-6-(1-methylethenyl)-2-cyclohexen-1-yl]-5-pentyl-, (1R-trans)-, Cannabinolic acid A (CBNA), Dronabinol (−8.1 kcal/mol) and Cannabigerolic acid (CBGA) to the protein.

Also, for bacteria to effectively colonize host tissues, they must react to and eliminate many antimicrobial chemicals made by the host, such as nitric oxide (NO). NO inhibits the growth of bacteria explicitly by going after proteins with Fe-S clusters [28]. NO was a signaling chemical involved in several pathways, including vasodilation and infection response, depending on subcellular concentrations [29]. Recent research has shown that the Rrf2-type transcriptional repressor; NsrR in *N. gonorrhoeae* can identify the presence of NO and regulate the genes expression that was involved in NO metabolism. The protein found to perform this function had a 2Fe-2S cluster [30]. This suggests that these proteins can ideally be targeted as the potential drug target. Molecular docking was performed by using Autodock Tools for 16 cannabinoids, of which 9 were from literature studies and 7 were obtained from fingerprint searches in the NPASS database as presented in Table 2. The binding energies of optimally docked molecules ranged from −8.0 to −11.71 kcal/mol [31]. As shown from molecular docking of the cannabinoids within the *N. gonorrhoeae*, 2Fe-2s cluster binding domain-containing protein, the 5 ligands based on the optimal binding energies, were highlighted as the most promising cannabinoids; 1,3-Benzenediol, 2-[3-methyl-6-(1-methylethenyl)-2-cyclohexen-1-yl]-5-pentyl-, (1R-trans)- had the highest binding affinity of −8.4 kcal/mol followed by Ferruginene C which was the derivative of cannabinoids identified from NPASS (−8.3 kcal/mol) and Dronabinol (−8.1 kcal/mol), which was derivatives of cannabinoids identified from the NPASS database. All of the cannabinoids were found to consistently dock at the same active binding site as illustrated in Figure 1. Building on this, Figure 2 presents the predicted binding pocket of the 2Fe-2S iron–sulfur cluster protein and illustrates the docking poses of these top-ranked compounds, demonstrating their interactions within a conserved active site. The visualization underscores that all ligands consistently docked at the same predicted active region, suggesting a stable and well-defined interaction interface that supports their potential as inhibitors of this critical bacterial protein.

Two phytocannabinoids were also identified as an intriguing phyto ligands CBGA and CBNA with the binding affinity of (−8.0 kcal/mol) and (−8.1 kcal/mol), respectively. These phytochemicals were found to be most abundant in cannabis varieties as a result of interbred between different plants [26]. Both are non-psychoactive with low cannabinoid (CB) receptor activity, but their potent antioxidant and anti-inflammatory characteristics make them excellent candidates for treating inflammatory conditions. Moderate inhibition was observed for the rest of the cannabinoids (binding affinity ranges from −6.6 to −7.9 kcal/mol) as presented in Table 3. Several phytoligands were found to exhibit high binding affinity scores. This means that the ligand can cause detrimental to the bio cellular processes of the Fe-S *N*. *gonorrhoeae* bacterial protein without using up too much energy. This finding aligns with the importance of secondary plant metabolites (SPMs), which also includes phytocannabinoids, which are recognized for their capacity to regulate cellular functions while controlling energy usage [32]. SPMs play significant roles in regulating oxidative stress and redox, which indicates that they exert hermetic effect where it depends on the circumstances and amount and the ability of it to cause variety of responses from cell growth to cell death [33]. Importantly, mitochondria play a crucial role in the plant’s reaction to stress, and they have the ability to absorb free radicals, which have been well documented as functioning as overall reservoirs for reactive oxygen species (ROS) as well as hydrogen peroxide [34,35]. Additionally, this occurs through the respiration process that has the ability to eliminate oxygen and potentially generate free radicals [36]. Recent research has revealed that hyperoxia, which refers to an excessive amount of oxygen exposure, has the ability to disrupt Fe-S-containing proteins. This disruption mostly impacts the electron transport chain (ETC), rendering it very susceptible to damage [37].

Owing to the current attention and breakthrough in Fe-S biology, which was highly probable to observe an increasing number of clinically utilized medicine increasing in the near future to emerge as compounds that interfere with Fe-S centers [38]. Targeting Fe-S clusters might be a promising technique for drug development. Considering Fe-S centers are required for cell longevity, it was conceivable that these cluster-targeting drugs paired with intrinsic impairments in their biogenesis can have a synergistic or cumulative effect [38]. These targeting methods based on the degradation of the Fe-S and/or disintegration after drug regimen can result in a stationary impact, triggering metabolic pause in pathogens [39]. This is due to the fact that many of this clusters have been depicted as “repairable” and the biogenesis of the iron–sulfur may be temporarily inhibited [40,41]. Hence, drugs that target Fe-S clusters may not necessarily result in the quick mortality of cells. When contemplating the combination in targeting these clusters with other cell-killing techniques, this factor will be significant. Thus, our findings suggest a significant finding against iron–sulfur cluster of *N*. *gonorrhoeae* and the potential use of these cannabinoids as anti-infectious medications.

### 3.3. Evaluation of Protein Ligand Interaction of the Identified Cannabinoids

The protein—ligand interaction molecular visualization was performed by using pyMOL. Further interactions of the proteins are evaluated by using PLIP. The hydrophobic interactions and hydrogen bonding are explored through PLIP. PLIP gets rid of the hydrophobic interactions between rings that are linked by π stacking as the stacking already involves hydrophobic interactions. The hydrophobic interactions considered only ligand atom with the shortest distance if the atom of the ligand interacts with multiple binding site atoms in a single residue [18]. These interactions are not supported in PLIP covalent bonds; weak hydrogen linkages including carbon atoms, halogen–water hydrogen bridges, and higher degree water bridges [18]. Our observation from the molecular docking resulted most of the ligands atom forms hydrogen bond primarily with Phe331. Further details are described in Supplementary Table S1.

#### 3.3.1. (+)-Cannabidiol: 1,3-Benzenediol, 2-[3-methyl-6-(1-methylethenyl)-2-cyclohexen-1-yl]-5-pentyl-, (1R-Trans)-

The receptor of CB1 was the main cannabinoid receptor in the brain, and it was increasingly being studied as a potential pharmacological target for a wide range of conditions, including nausea, cachexia, obesity, pain, stiffness, neurodegenerative illnesses, and mood and substance addiction problems [26]. Cannabis produces (−)-CBD, although CBD can also exist as the (+)-CBD enantiomer and was potentially found to inhibit cannabinoid CB1 receptors significantly more potently and activates sphingosine-1-phosphate receptors in an enantiomer-specific way [27].

**Figure 3:**
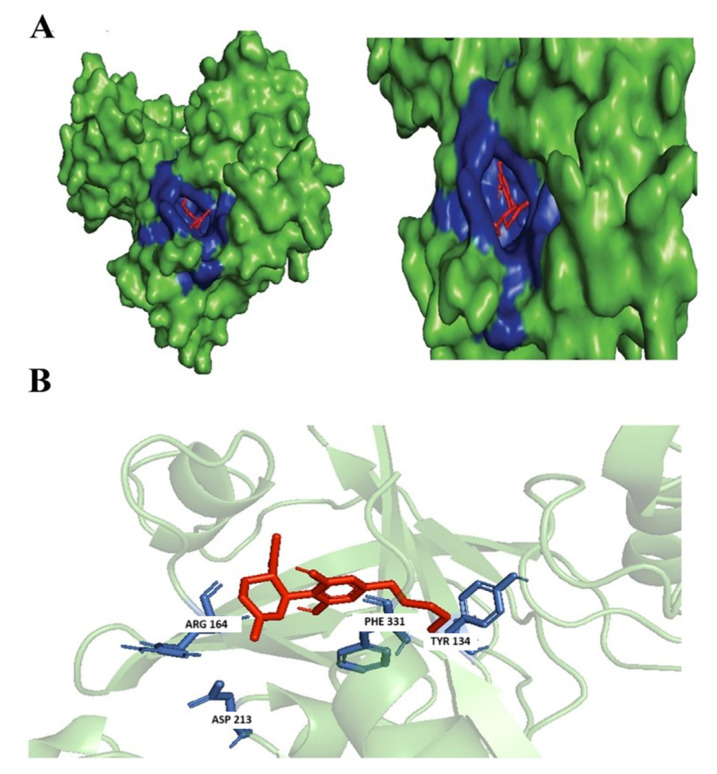
Docking of 1,3-Benzenediol, 2-[3-methyl-6-(1-methylethenyl)-2-cyclohexen-1-yl]-5-pentyl-, (1R-trans)-. (**A**) Blue-colored region represents non-polar interactions; yellow region represents hydrogen bond (polar interactions). (**B**) Ligand interactions with labeled residues with blue amino acids (AA) chain; non-polar interactions and yellow; hydrogen bonds.

Not many studies explored the potential of the enantiomers of CBD as antibacterial agents; however, there were studies associated with its property as an anti-inflammatory by reducing the activation of encephalitogenic T cells [7]. In our study, targeted 1,3-Benzenediol, 2-[3-methyl-6-(1-methylethenyl)-2-cyclohexen-1-yl]-5-pentyl-, (1R-trans)- one of the derivates of (+)-CBD, demonstrated the highest binding affinity at the targeted *N. gonorrhoeae* protein’s active binding site. The protein–ligand complex interacts primarily with Phe331, Asp213, Arg164, and Tyr134 forming hydrophobic interactions, but no hydrogen bond was formed (Figure 3).

#### 3.3.2. Dronabinol

**Figure 4:**
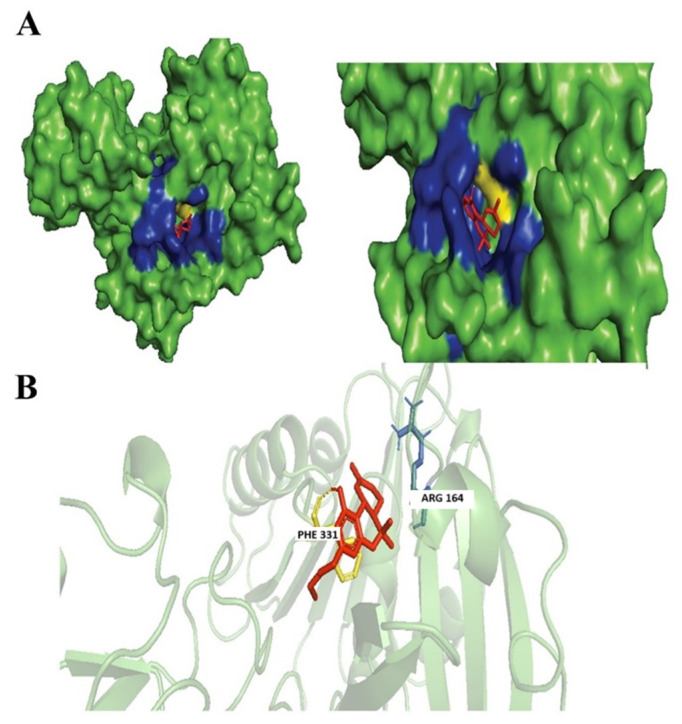
Docking of Dronabinol. (**A**) Blue colored region represents non-polar interactions; the yellow region represents hydrogen bonds (polar interactions). (**B**) Ligand interactions with labeled residues with blue amino acids (AA) chain; non-polar interactions and yellow; hydrogen bonds.

Dronabinol is an isomer of THC, the dominant and most active isomer found in the *Cannabis sativa* L. plant, with potential anti-emetic, analgesic, and hunger-stimulating properties [42]. Based on our findings Dronabinol was also found to have similarities of ≥99% with Tetrahydrocannabivarin with a chemical formula of C21H30O2. Dronabinol and nabilone were licensed in the 1980s by the Food and Drug Administration (FDA) of the United States for the treatment of anorexia caused by weight loss in people with acquired immunodeficiency syndrome (AIDS) and for the prevention of nausea and vomiting caused by chemotherapy [42]. Dronabinol was found to exhibit antimicrobial activities through the successful inhibition of *Aspergillus niger*, *Candida albicans*, *Staphylococcus aureus*, *Pseudomonas aeruginosa*, *and Escherichia coli* [43]. Dronabinol was observed to form hydrophobic interactions with Arg164 and hydrogen bonds with Phe331 residue within our targeted *N*. *gonorrhoeae* protein (Figure 4).

#### 3.3.3. Cannabinolic Acid A (CBNA)

**Figure 5:**
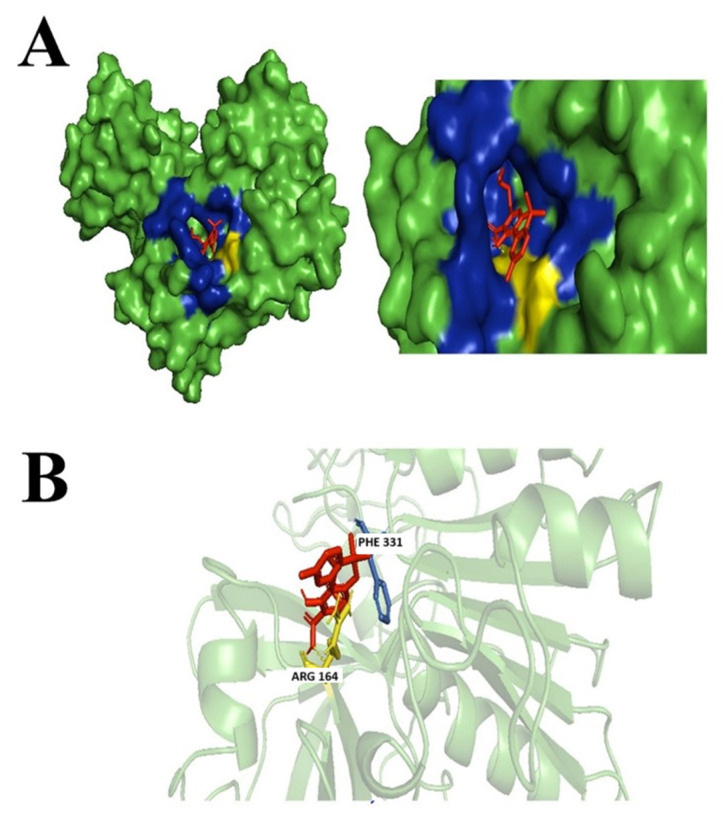
Docking of CBNA. (**A**) Blue colored region represents non-polar interactions; the yellow region represents hydrogen bonds (polar interactions). (**B**) Ligand interactions with labeled residues with blue amino acids (AA) chain; non-polar interactions and yellow; hydrogen bonds.

CBNA is one of the natural derivates of THC as well as the precursor molecule of cannabinol (CBN) and is non-intoxicating [13]. CBNA can also be synthesized from tetrahydrocannabinol acid (THCA); by aromatization employing, selenium dioxide combined with trimethylsilyl polyphosphate as a catalyst in chloroform, a key component of the cannabis plant is produced [44]. CBNA is found to potentially act as an antibacterial and, in a recent study, was found to inhibit *N. gonorrhoeae* ATCC 19424 having the lowest inhibitory concentration (MIC) of μg·mL^−1^ of 0.25 to 4 [13]. Similarly found, CBNA was found to successfully inhibit our targeted drug target protein with a considerably high binding affinity score forming a hydrogen bond with Arg164 residue and hydrophobic interaction with Phe331 (Figure 5). As observed, the interaction found is similar to Dronabinol.

#### 3.3.4. Cannabigerolic Acid (CBGA)

**Figure 6:**
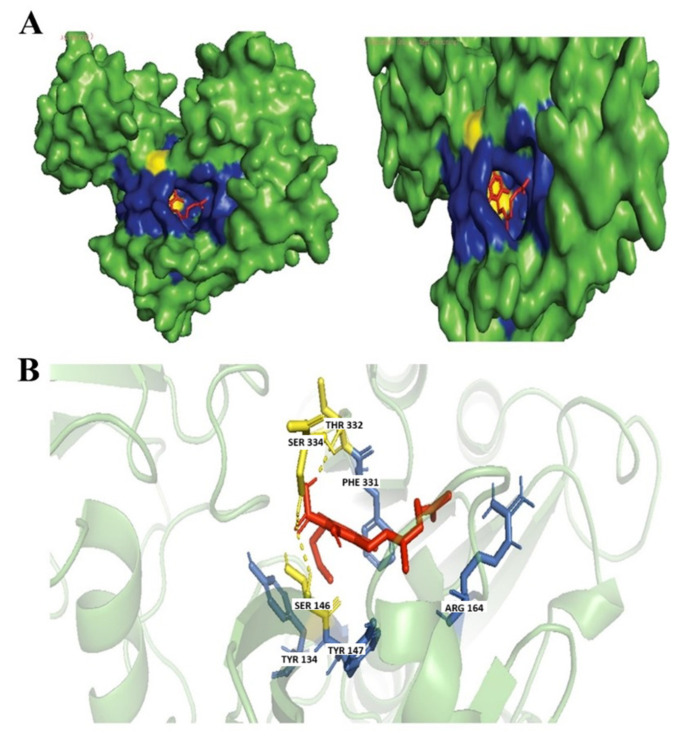
Docking of CBGA. (**A**) Blue colored region represents non-polar interactions; the yellow region represents hydrogen bonds (polar interactions). (**B**) Ligand interactions with labeled residues with blue amino acids (AA) chain; non-polar interactions and yellow; hydrogen bonds.

CBGA is a precursor biosynthetic of THC, the major psychoactive component of the cannabis plant. It is a diterpenoid, a dihydroxybenzoic acid at which the hydrogen at position 3 of olivetolic acid has been replaced with a geranyl group, a polyketide, a resorcinol, and a phytocannabinoid [13]. CBGA is a well-known secondary cannabinoid with anti-inflammatory and antiviral properties inhibiting SARS-CoV2 infection [36]. CBGA also has potent antibacterial properties as CBGA was found to successfully inhibit *N. gonorrhoeae* ATCC 19424 with a MIC of μg·mL^−1^ of 1 to 2 [13]. Another study found the treatment of CBGA was also found effective to reduce the bacterial content of dental plaque [45]. Based on our molecular docking analysis, CBGA was found to form a hydrogen bond with Ser146, Ser334, and Thr332 residues and hydrophobic interactions with Arg164, Phe331, Tyr134, and Tyr147 (Figure 6).

### 3.4. Evaluation of Protein Ligand Interaction of the Cannabinoid’s Derivatives

#### Ferruginene C

**Figure 7:**
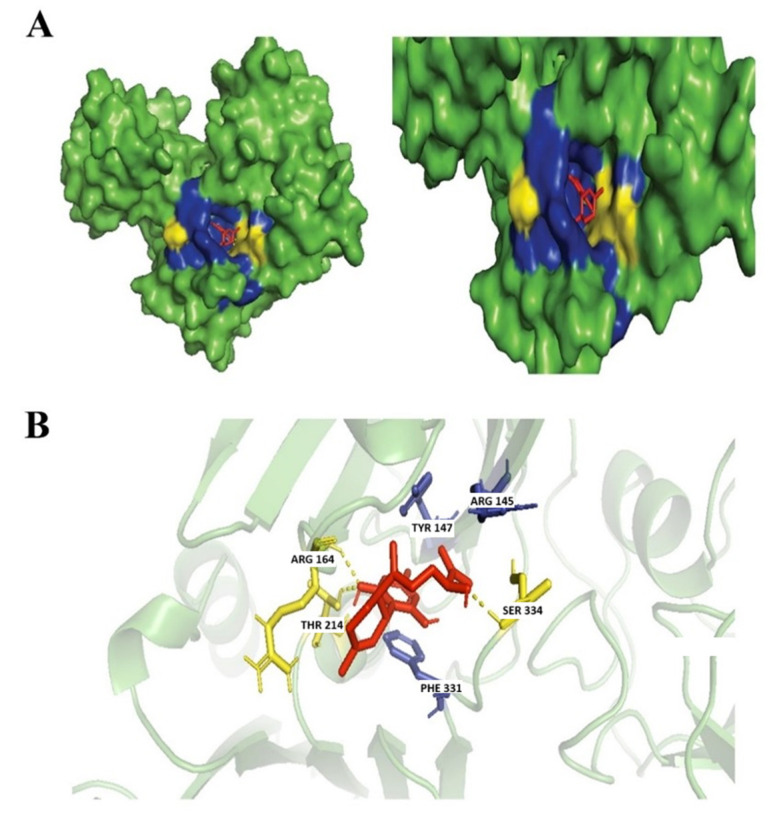
Docking of Ferruginene C. (**A**) Blue colored region represents non-polar interactions; yellow region represents hydrogen bond (polar interactions). (**B**) Ligand interactions with labeled residues with blue amino acids (AA) chain; non-polar interactions and yellow; hydrogen bonds.

Ferruginene C is a sesquiterpenoid. It has a role as a metabolite and a phytochemical that is found in *Rhododendron ferrugineum* [46]. The antimicrobial property of this compound has not yet been reported but our findings suggest that Ferruginene C can be further explored as a potential antimicrobial against *N. gonorrhoeae* with a high binding affinity score Table 2. In our studies, the interaction of the ligand-protein was observed to form a hydrogen bond with Arg164, Thr214, and Ser334 residues and hydrophobic interactions dominantly with Phe331, Tyr147, and Arg145 (Figure 7).

### 3.5. Drug-likeness and Pharmacokinetics Profile Assessment of the Identified Cannabinoids and Derivatives

The identified cannabinoids and derivatives were further assessed for their drug-likeness property to ADME prediction to evaluate their pharmacokinetic and toxicity property. Violation of Lipinski rules of 5 and BOILED-EGG prediction is achieved by using SWISSADME. Further BOILED-EGG analysis results are provided in the Supplementary Material Figure S1A–E. Based on the evaluation of the identified, most of the cannabinoids showed no violation when assessed based on Lipinski rules of 5 except for CBGA, Dronabinol, and 1,3-Benzenediol, 2-[3-methyl-6-(1-methylethenyl)-2-cyclohexen-1-yl]-5-pentyl-, (1R-trans)- which have 1 violation for MLOGP > 4.15, which is still considerate. The ADME prediction result illustrated in Table 4 indicates that most of the cannabinoids showed high gastrointestinal (GI) absorption and are not likely permeable to the human Blood–Brain Barrier (BBB) except for 1,3-Benzenediol, 2-[3-methyl-6-(1-methylethenyl)-2-cyclohexen-1-yl]-5-pentyl-, (1R-trans)- and Ferruginene C. Based on the analysis, most of the cannabinoids are not suitable for oral bioavailability due to high lipophilicity and poorly soluble except for Ferruginene C.

To assess the safety and effectiveness of the compound, comparisons to commonly used antibiotics (Azithromycin, Cefixime, Ceftriaxone, and Ciprofloxacin) in treating *N. gonorrhoeae* are required. The Lipinski analysis revealed that Azithromycin and Ceftriaxone exhibited two violations, specifically exceeding the thresholds for molecular weight (MW > 500) and polar surface area (NorO > 10). The findings were tabulated in Table 5. Similarly, the ADME and toxicity analysis is also performed for the antibiotics and tabulated in Table 6.

The ADME and toxicity test of the identified compounds were assessed based on the pkCSM theory and the result is illustrated in Table 7 [47]. The majority of drugs taken orally are assimilated through the intestinal mucosa. Caco-2 monolayers are widely used as an in vitro model to investigate and predict the absorption of medications in humans. These cells are derived from a human colon adenocarcinoma and, upon differentiation, form polarized monolayers that mimic the intestinal epithelium [48]. Based on a study suggested that the overall ranking for Caco-2 permeability is as follows; 0–20% as poor, 20–70% as moderate, and 70–100% as well absorbed chemicals are those with Papp 1 × 10^−6^ cm/s, between 1–10 × 10^−5^ cm/s, and >10 × 10^−6^ cm/s, respectively [49]. High Caco-2 permeability would equate to values greater than 0.9 in the pkCSM predictive model. Hence, moderate permeability through CaCo-2 cells was observed displayed for CBNA and CBGA while high permeability was observed for 1,3-Benzenediol, 2-[3-methyl-6-(1-methylethenyl)-2-cyclohexen-1-yl]-5-pentyl-, (1R-trans)-, Dronabinol and Ferruginene C. As for the antibiotics, moderate permeability is observed for Azithromycin and Ciprofloxacin, while poor permeability is seen for Cefixime and Ceftriaxone.

The organ toxicity was assessed through ProTox-II. The probability scores represent the model’s confidence in classifying the compound as active (toxic) or inactive (non-toxic). Based on the analysis, across the five compounds, the toxicity profiles vary for the targeted organ. Hepatotoxicity and neurotoxicity were observed as active only for the third compound, Ferruginene C while nephrotoxicity was classified as active for the CBNA and CBGA compounds. Mutagenicity was predicted as inactive for all compounds, indicating a low likelihood of mutagenic potential across the dataset. This suggests that none of the compounds exhibit broad or consistent high-risk toxicity profiles. Hence, such profiles indicate that these compounds retain potential for therapeutic development provided that the predicted toxicities are further assessed and mitigated through targeted structural optimization and preclinical validation.

In contrast, all of the antibiotics showed no heptotoxicity. Neurotoxicity and nephrotoxicity were variable, with some antibiotics predicted active; Azithromycin, Cefixime and Ciprofloxacin. While mutagenicity was inactive for Azithromycin and Ceftriaxone, but active for Cefixime and Ciprofloxacin. This profile indicates that the toxicity varied in other organs. Overall, the identified compounds demonstrate generally safer toxicity profiles compared to the antibiotics, supporting their potential for drug development with further testing and validations.

P-glycoprotein is the ATP-binding cassette (ABC) transporter that has been studied the most. It acts as a biological barrier by pushing toxins and xenobiotics out of cells. It also plays a big role in how drugs are absorbed and disposed of. Because of where it is found, P-glycoprotein seems to have a bigger effect on stopping cells from taking in drugs from the bloodstream into the brain and from the intestinal lumen into epithelial cells than on making it easier for drugs to get out of hepatocytes and renal tubules and into the space next to them [50]. Based on the findings, it is indicated that almost all of the cannabinoids are Pgp substrates except for Dronabinol. While for the antibiotics on Azithromycin is recognized as both Pgp I and Pgp II inhibitors. The volume of distribution (VDss) is the amount of fluid that “seems” to be needed to retain all of the drugs in the body at the same concentration as in plasma (or blood) [51]. The more the VD increases, the more of the drug is found in tissue instead of plasma. Based on the pkCSM model, the VDss value is low if below 0.71 L/kg which translates to (log VDss < −0.15) and considered high if above 2.81 L/kg correlates to (log VDss > 0.45). Our analysis showed low VD values for CBNA and CBGA while moderate for the rest of the cannabinoids. Low VS values were observed for all of the antibiotics. The BBB possess unique properties to vascularize the central nervous system (CNS), which allow them to closely regulate the transit of ions, chemicals, and cells between the blood and the brain [48]. The drug’s ability to enter the brain is an important factor to think about if you want to reduce side effects and toxicity or improve the effectiveness of drugs whose pharmacological effects happen in the brain. Though it can be difficult to determine how to measure the BBB. A more direct metric is the blood–brain permeability-surface area product (log P). Most of the cannabinoids including Ferruginene C are considered to penetrate the CNS with a logPS value > −2.5. As for the antibiotics, only Ciprofloxacin is seen as capable of penetrating the CNS.

The cytochrome P450 (CYP) enzymes are membrane-bound hemoproteins that are essential for xenobiotic detoxification, cellular metabolism, and homeostasis [49]. CYPs have the ability to regulate pharmacological action, safety, bioavailability, and resistance through metabolism in both metabolic organs and local sites of action [52]. Because of their ability to either inhibit or promote the CYP enzyme system, several chemotherapeutic medications might result in drug interactions. 1,3-Benzenediol, 2-[3-methyl-6-(1-methylethenyl)-2-cyclohexen-1-yl]-5-pentyl-, (1R-trans)- and Dronabinol elucidated the ability to inhibit CYP1A2, CYP2C19, CYP2C19, and CYP3A4 and act as CYP3A4 substrate, while CBGA resulted in no inhibition of any of the P450 cytochromes. While all of the antibiotics are not found as either inhibitors or substrates of the CYP.

On the basolateral (blood) side of proximal tubule cells, organic cation transporter 2 (OCT2) is mostly expressed as a renal uptake transporter. It is essential for the disposal and renal clearance of cationic medications and chemicals that are endogenous. The substrates of OCT2 have the potential to generate pharmacokinetic adverse effects, yet none of the discovered compounds are OCT2 substrates. The Salmonella typhimurium reverse mutation assay (AMES test) is a rapid bacterial test that evaluates presence of carcinogens by observing the changes in bacteria [53]. The result indicated that all cannabinoids are not carcinogens based on the AMES test. The findings also suggested that none of the antibiotics are carcinogens. Lastly, throughout the drug development process, ventricular arrhythmias are one of the most common undesirable side events that result in drug failure. This is mostly due to the drug’s inability to inhibit the cardiac potassium channel of the human ether-à-go-go-related gene (hERG) [54]. The result indicated that mostly all cannabinoids are not identified as hERG I and II inhibitor except for Dronabinol that are likely to act as an hERG II inhibitor. The hepatotoxicity test also indicated that all the cannabinoids are unlikely to disrupt normal liver function. While all the antibiotics indicated potential in disrupting the liver function.

## 4. Conclusions

Cannabinoids have the potential to be examined as possible antibiotics for the treatment of *N. gonorrhoeae*, according to the findings of this study. Given the availability of *C. sativa* strains producing considerable quantities of non-psychotropic cannabinoids, this plant is an exciting source of antibacterial medications to combat the problem of *N. gonorrhoeae* and other negative-gram pathogens’ multidrug resistance. Although iron–sulfur (FeS) clusters are one of the oldest types of bio-inorganic cofactors, their roles in Neisseria gonococcal pathogenesis have received little study. Our findings indicate that the identified cannabinoids; 1,3-Benzenediol, 2-[3-methyl-6-(1-methylethyl)-2-cyclohexen-1-yl] -5-pentyl-, (1R-trans)-, Ferruginene C, which is the derivative of 7-Hydroxycannabidiol, Dronabinol, CBNA, and CBGA can be used as inhibitors against FeS cluster binding domain-containing protein and are hence a prospective therapeutic target for *N. gonorrhoeae*. According to our research, some cannabinoids could inhibit the Fe-S cluster *N. gonorrhoeae* bacterial protein and therefore treat the infections. In addition, it gives a safer alternative to synthetic antibiotics to lower the risk of the emergence of drug resistance. Additional in vivo experimental is required to develop effective drugs to combat against *N. gonorrhoeae*.

## Figures and Tables

**Table 1:** Compounds based on literature findings.

Cannabinoids	Pubhem ID	Chemical Formula	Weight (g/mol)
Cannabidiol	644019	C_21_H_30_O_2_	314.46
7-Hydroxycannabidiol	11301963	C_21_H_30_O_3_	330.46
Cannabidivarin	11601669	C_19_H_26_O_2_	286.41
Cannabidiolic acid	160570	C_22_H_30_O_4_	358.4
Tetrahydrocannabivarin (THCV)	93147	C_19_H_26_O_2_	286.41
Tetrahydrocannabivarinic acid (THCVA)	59444416	C_20_H_26_O_4_	330.42
Cannabinolic acid A (CBNA)	3081990	C_22_H_26_O_4_	354.44
Cannabigerol (CBG)	5315659	C_21_H_32_O_2_	316.48
Cannabigerolic acid (CBGA)	6449999	C_22_H_32_O_4_	360.49

**Table 2:** Compound identified having similarities of ≥99% against the cannabinoid’s analog.

Searched Compound	Identified Compound	NPASS ID	PubChem ID	Chemical Formulae	Molecular Weight
Tetrahydrocannabivarin (THCV)	Dronabinol	NPC96940	16078	C_21_H_30_O_2_	314.22
Cannabidiol	1,3-Benzenediol, 2-[3-methyl-6-(1-methylethenyl)-2-cyclohexen-1-yl]-5-pentyl-, (1R-trans)-	NPC186385	36688143	C_21_H_30_O_2_	314.22
7-Hydroxycannabidiol	Ferruginene C	NPC319803	52951888	C_22_H_30_O_3_	342.22
Tetrahydrocannabivarinic acid (THCVA)	Δ9-Tetrahydrocannabinolic Acid A	NPC150928	98523	C_22_H_30_O_4_	358.21
Cannabigerol (CBG)	Grifolin	NPC12640	5372312	C_22_H_32_O_2_	328.4
	Sesquicannabigerol	NPC99836	54669855	C_26_H_40_O_2_	384.3
Cannabigerolic acid (CBGA)	Grifolic Acid	NPC180261	9976563	C_23_H_32_O_4_	372.23
Cannabidivarin	1,3-Benzenediol, 2-[3-methyl-6-(1-methylethenyl)-2-cyclohexen-1-yl]-5-pentyl-, (1R-trans)-	NPC186385	36688143	C_21_H_30_O_2_	314.22
	Cannabidiol	NPC299568	644019	C_21_H_30_O_2_	314.46

**Table 3:** Docking binding affinity of sixteen cannabinoids to the 2Fe-2S iron–sulfur cluster binding domain-containing protein (accession ID: NGFG RS03485).

Compounds	Binding Affinity (kcal/mol)
Tetrahydrocannabivarin (THCV)	−7.9
Cannabidiolic acid	−7.8
Cannabidiol	−7.5
Cannabinolic acid A (CBNA)	−8.1
Grifolin	−7.7
Ferruginene C	−8.3
1,3-Benzenediol, 2-[3-methyl-6-(1-methylethenyl)-2-cyclohexen-1-yl]-5-pentyl-, (1R-trans)-	−8.4
Sesquicannabigerol	−6.6
Grifolic acid	−7.9
delta(9)-Tetrahydrocannabinolic acid	−7.6
Dronabinol	−8.1
Cannabigerol (CBG)	−6.8
Cannabigerolic acid (CBGA)	−8.0
7-Hydroxycannabidiol	−7.4
Tetrahydrocannabivarinic acid (THCVA)	−7.9
Cannabidivarin	−7.8

**Table 4:** Drug-likeness assessment of the identified cannabinoids and its derivative.

Cannabinoids	Molecular Weight g/mol	Lipinski Rules of 5 (ROF)	Chemical Formula	Log Po/w (WLOGP)	Log S (SILI- COS-IT)	BBB Permeation	Gastrointestinal (GI) Absorption	PGP Substrate	Suitable for Oral Bioavailability
Cannabinolic acid A (CBNA)	354.44	Yes; 0 violation	C_22_H_26_O_4_	5.32	−6.84	No	High	No	No. High lipophilicity and poorly soluble
Cannabigerolic acid (CBGA)	360.49	Yes; 1 violation: MLOGP > 4.15	C_22_H_32_O_4_	5.76	−5.14	No	High	No	No. High lipophilicity, too flexible and moderately soluble
Dronabinol	314.22	Yes; 1 violation: MLOGP > 4.15	C_21_H_30_O_2_	5.74	−5.93	Yes	High	No	No. High Lipophilicity
1,3-Benzenediol, 2-[3-methyl-6-(1-methylethenyl)-2-cyclohexen-1-yl]-5-pentyl-, (1R-trans)-	314.22	Yes; 1 violation: MLOGP > 4.15	C_21_H_30_O_2_	5.85	−5.41	Yes	High	No	No. High Lipophilicity
Ferruginene C	342.22	Yes; 0 violation	C_22_H_30_O_3_	5.12	−4.51	Yes	High	No	Yes. Moderately Soluble

**Table 5:** Drug-likeness assessment of commonly used antibiotics to treat *N. gonorrhoeae*.

Antibiotic	Molecular Weight g/mol	Lipinski Rules of 5 (ROF)	Chemical Formula	Log Po/w (WLOGP)	Log S (SILI- COS-IT)	BBB Permeation	Gastrointestinal (GI) Absorption	PGP Substrate	Suitable for Oral Bioavailability
Azithromycin	748.98	No; 2 violations: MW > 500, NorO > 10	C_38_H_72_N_2_O_12_	1.52	−2.22	No	Yes	No	No. High molecular weight, high polarity and insoluble
Cefixime	453.45	Yes; 1 violation: NorO > 10	C_16_H_15_N_5_O_7_S_2_	−0.92	−0.75	No	No	No	No. Too polar
Ceftriaxone	554.58	No; 2 violations: MW > 500, NorO > 10	C_18_H_18_N_8_O_7_S_3_	−1.98	−2.08	No	No	No	No, high molecular weight and high polarity
Ciprofloxacin	331.34	Yes; 0 violation	C_17_H_18_FN_3_O_3_	1.18	−3.5	No	High	Yes	Yes. Highly Soluble

**Table 6:** ADMET analysis for commonly used antibiotics to treat *N. gonorrhoeae*.

Antibiotic		Azithromycin	Cefixime	Ceftriaxone	Ciprofloxacin
Absorption	Water solubility (log mol/L)	−3.144	−2.73	−2.843	−2.692
	Caco2 permeability (log Papp in 10-6 cm/s)	0.119	−0.392	−0.457	0.675
	Intestinal absorption (human) (% Absorbed)	0.119	14.687	12.93	98.704
	Skin Permeability (log Kp)	−2.735	−2.735	−2.735	−2.735
	P-glycoprotein substrate	Yes	Yes	Yes	Yes
	P-glycoprotein I inhibitor	Yes	No	No	No
	P-glycoprotein II inhibitor	Yes	No	No	No
Distribution	VDss (human) (log L/kg)	1.179	−1.647	−2.286	−0.395
	Fraction unbound (human) (Fu)	0.71	0.543	0.365	0.646
	BBB permeability (log BB)	−1.74	−1.575	−2.092	−0.555
	CNS permeability (log PS)	−4.704	−4.038	−4.516	−2.957
Metabolism	CYP2D6 substrate	No	No	No	No
	CYP3A4 substrate	No	No	No	No
	CYP1A2 inhibitor	No	No	No	No
	CYP2C19 inhibitor	No	No	No	No
	CYP2C9 inhibitor	No	No	No	No
	CYP2D6 inhibitor	No	No	No	No
	CYP3A4 inhibitor	No	No	No	No
Excretion	Total Clearance (log mL/min/kg)	−0.404	0.076	−0.188	0.618
	Renal OCT2 substrate	No	No	No	No
Toxicity	AMES toxicity	No	No	No	No
	Max. tolerated dose (human) (log mg/kg/day)	1.088	1.534	1.099	0.771
	hERG I inhibitor	No	No	No	No
	hERG II inhibitor	No	No	No	No
	Oral Rat Acute Toxicity (LD50) (mol/kg)	2.352	1.947	2.319	2.661
	Oral Rat Chronic Toxicity (LOAEL) (log mg/kg_bw/day)	3.013	2.587	2.614	0.851
	Hepatotoxicity	Yes	Yes	Yes	Yes
	Skin Sensitisation	No	No	No	No
	T.Pyriformis toxicity (log μg/L)	0.285	0.285	0.285	0.851
	Minnow toxicity (log mM)	0.285	3.613	3.523	1.71
Organ Toxicity	Hepatotoxicity	Inactive	Inactive	Inactive	Inactive
	Neurotoxicity	Active	Inactive	Inactive	Active
	Nephrotoxicity	Active	Active	Inactive	Active
	Mutagenicity	Inactive	Active	Inactive	Active

**Table 7:** ADME pharmacokinetic properties assessment and toxicity test of the cannabinoids and its derivative.

Ligand		Cannabinolic Acid A (CBNA)	1,3-Benzenediol, 2-[3-Methyl-6-(1-methylethenyl)-2-cyclohexen-1-yl]-5-pentyl-, (1R-Trans)-	Ferruginene C	Dronabinol	Cannabigerolic Acid (CBGA)
Property	Model Name	Predicted Value				
Absorption	Water solubility (log mol/L)	−3.473	−5.609	−4.585	−5.042	−3.515
	Caco2 permeability (log Papp in 10^−6^ cm/s)	0.664	1.227	1.411	1.539	0.588
	Intestinal absorption (human) (% Absorbed)	95.906	89.308	89.599	91.162	95.782
	Skin Permeability (log Kp)	−2.735	−2.784	−2.855	−2.669	−2.735
	P-glycoprotein substrate	Yes	Yes	Yes	No	Yes
	P-glycoprotein I inhibitor	No	No	No	Yes	No
	P-glycoprotein II inhibitor	No	Yes	No	No	Yes
Distribution	VDss (human) (log L/kg)	−1.623	0.771	0.493	0.977	−1.575
	Fraction unbound (human) (Fu)	0	0	0.078	0.005	0.074
	BBB permeability (log BB)	0.176	−0.074	−0.345	0.489	−0.911
	CNS permeability (log PS)	−1.853	−1.741	−0.345	−1.807	−2.269
Metabolism	CYP2D6 substrate	No	No	No	No	No
	CYP3A4 substrate	No	Yes	No	Yes	No
	CYP1A2 inhibitor	No	Yes	No	Yes	No
	CYP2C19 inhibitor	No	Yes	No	Yes	No
	CYP2C9 inhibitor	Yes	No	No	No	No
	CYP2D6 inhibitor	No	No	No	No	No
	CYP3A4 inhibitor	No	Yes	Yes	No	No
Excretion	Total Clearance (log ml/min/kg)	0.663	1.267	1.198	0.881	1.278
	Renal OCT2 substrate	No	No	No	No	No
Toxicity	AMES toxicity	No	No	No	No	No
	Max. tolerated dose (human) (log mg/kg/day)	0.585	−0.18	−0.062	−0.207	−0.073
	hERG I inhibitor	No	No	No	No	No
	hERG II inhibitor	No	No	No	Yes	No
	Oral Rat Acute Toxicity (LD50) (mol/kg)	2.71	2.55	2.727	2.62	2.588
	Oral Rat Chronic Toxicity (LOAEL) (log mg/kg_bw/day)	1.971	2.639	2.443	1.885	2.057
	Hepatotoxicity	No	No	No	No	No
	Skin Sensitisation	No	Yes	No	No	No
	T.Pyriformis toxicity (log µg/L)	0.285	1.998	1.774	1.919	0.285
	Minnow toxicity (log mM)	−1.328	−0.57	−0.198	−0.917	−0.825
Organ Toxicity	Hepatotoxicity	Inactive	Inactive	Active	Inactive	Inactive
	Neurotoxicity	Inactive	Inactive	Active	Inactive	Inactive
	Nephrotoxicity	Active	Inactive	Inactive	Inactive	Active
	Mutagenicity	Inactive	Inactive	Inactive	Inactive	Inactive

## Data Availability

Not applicable.

## Supplementary Materials

The following supporting information can be downloaded at: https://www.mdpi.com/article/10.3390/biology14091272/s1, Figure S1A: A: BOILED-Egg analysis indicates that 1,3-Benzenediol, 2-[3-methyl-6-(1-meth-ylethenyl)-2-cyclohex-en-1-yl]-5-pentyl-, (1R-trans)- exhibits high predicted gastrointestinal absorption, effective blood–brain barrier penetration, and is not a substrate of P-glycoprotein; Figure S1B: BOILED-Egg analysis indicates that dronabinol exhibits high predicted gastrointestinal absorption, effective blood–brain barrier penetration, and is not a substrate of P-glycoprotein; Figure S1C: BOILED-Egg analysis indicates that CBNA exhibits high predicted gastrointestinal absorption, effective blood–brain barrier penetration, and is not a substrate of P-glycoprotein; Figure S1D: BOILED-Egg analysis indicates that Cannabigerolic acid (CBGA) exhibits high predicted gastrointestinal absorption, is unlikely to cross the blood–brain barrier, and is not a substrate of P-glycoprotein; Figure S1E: BOILED-Egg analysis indicates that Ferruginene C exhibits high predicted gastrointestinal absorption, limited blood–brain barrier penetration, and is not a substrate of P-glycoprotein; Table S1: Protein-Ligand Interactions.

## Abbreviations

The following abbreviations are used in this manuscript:

ABC	ATP-binding cassette
ADT	AutoDock Tools
ADME	Absorption, Distribution, Metabolism, Excretion
AIDS	Acquired Immunodeficiency Syndrome
AMR	Antimicrobial Resistance
BBB	Blood–Brain Barrier
BOILED-Egg	Brain or Intestinal EstimateD permeation method
BSTIs	Bacterial Sexually Transmitted Infections
CB	Cannabinoid
CBN	Cannabinol
CBNA	Cannabinolic Acid A
CBGA	Cannabigerolic Acid
CNS	Central Nervous System
CYP	Cytochrome P450
ETC	Electron Transport Chain
ESC	Extended-Spectrum Cephalosporins
FDA	Food and Drug Administration
Fe-S	Iron–sulfur Proteins
FeS	Iron–sulfur
FUR	Ferric Uptake Regulator
GI	Gastrointestinal
hERG	Human Ether-à-Go-Go-Related Gene
HIA	Human Intestinal Absorption
MIC	Lowest Inhibitory Concentration
NO	Nitric Oxide
NPs	Natural Products
NPASS	Natural Product Activity and Species Source
PLIP	Protein-Ligand Interaction Profiler
Ro5	Rule-of-Five
ROS	Reactive Oxygen Species
SPMs	Secondary Plant Metabolites
STD	Sexually Transmitted Disease
SWISSADME	Swiss Institute of Bioinformatics ADME prediction tool
THC	Δ-9-Tetrahydrocannabinol
THCA	Tetrahydrocannabinol Acid
VDss	Volume of Distribution
3D	3-Dimensional
