# Supplementary material for: Investigating the Antimicrobial Efficacy of Cannabinoids and Their Derivatives Against Neisseria Gonorrhoeae by Computational Analysis
Source: Biology (Basel). 2025 Sep 15;14(9):1272. doi: 10.3390/biology14091272 (PMC12467043; doi:10.3390/biology14091272)
Supplement: Supplementary file 1 [file biology-14-01272-s001.zip › biology-3786638-supplementary.pdf]

Supplementary Material

Investigating the Antimicrobial Efficacy of Cannabinoids and Their Derivatives Against Neisseria Gonorrhoeae by Computational Analysis

Umairah Natasya Mohd Omeershffudin[1], Zakirah Zainal Abidin[1], Zaw Myo Hein[2], Che Mohd Nasril Che Mohd Nassir[3], Ebrahim Nangarath Kottakal Cheriya[4], Suresh Kumar[5]*, Muhammad Danial Che Ramli[5]*

1 Schools of Graduate Studies, Post Graduate Centre, Management and Science University, University Drive, Off Persiaran Olahraga, Section 13, 40100 Selangor, Malaysia.

2 Department of Basic Medical Sciences, College of Medicine, Ajman University, P.O.BOX:346 Ajman, United Arab Emirates.

3 Department of Anatomy and Physiology, School of Basic Medical Sciences, Faculty of Medicine, University Sultan Zainal Abidin, 20400 Kuala Terengganu, Terengganu, Malaysia.

4 Department of Physiology, International Medical School, Management and Science University, Seksyen 13, 40100, Shah Alam, Selangor, Malaysia.

5 Faculty of Health and Life Sciences, Management and Science University, Seksyen 13, 40100, Shah Alam, Selangor, Malaysia.

* Correspondence: sureshkumar@msu.edu.my, muhddanial_cheramli@msu.edu.my

Supplementary Table:

Supplementary Table S1: Protein-Ligand Interactions.

Protein	Hydrophobic Interactions					Hydrogen Bonds							
	Residue	AA	Distance	Ligand Atom	Protein Atom	Residue	AA	Distance H-A	Distance D-A	Donor Angle	Donor Atom	Acceptor Atom
1,3-Benzenediol, 2-[3-methyl-6-(1-methylethenyl)-2-cyclohexen-1-yl]-5-pentyl-, (1R-trans)-	134A	TYR	3.78	3131	1182	331A	PHE	3.02	3.74	132.19	3125 [O3]	3072 [O2]
	134A	TYR	3.99	3130	1184							
	164A	ARG	3.77	3116	1463							
	213A	ASP	3.9	3119	1925							
	331A	PHE	3.71	3111	3074							

	331A	PHE	3.57	3110	3073						
	331A	PHE	3.73	3131	3077						
	331A	PHE	3.46	3128	3075						
Ferruginene C	145A	ARG	3.59	3134	1279	164A	ARG	2.27	3.17	145.48	1458 [Nam]	3121 [O3]
	147A	TYR	3.35	3119	1306	214A	THR	2.22	2.9	128.17	1936 [O3]	3121 [O3]
	147A	TYR	3.84	3134	1308	214A	THR	2.48	2.9	106.12	3121 [O3]	1936 [O3]
	164A	ARG	3.71	3110	1463	332A	THR	3.28	4.09	142.37	3130 [O3]	3084 [O2]
	166A	ARG	3.3	3109	1496	334A	SER	2.03	2.95	147.54	3097 [Nam]	3130 [O3]
	331A	PHE	3.63	3119	3079							
	331A	PHE	3.44	3117	3078							
	331A	PHE	3.8	3114	3076							
Dronabinol	164A	ARG	3.38	3111	1462	331A	PHE	2.14	3.04	152.73	3129 [O3]	3072 [O2]
	331A	PHE	3.74	3114	3073							
Cannabinolic acid A (CBNA)	331A	PHE	3.72	3127	3076	147A	TYR	3.14	3.9	135.16	3133 [O3]	1311 [O3]
	331A	PHE	3.63	3129	3073	164A	ARG	2.43	3.18	129.7	1458 [Nam]	3133 [O3]
Cannabigerolic acid (CBGA)	134A	TYR	3.49	3130	1184	146A	SER	2.94	3.84	147.89	1292 [Nam]	3107 [O3]
	147A	TYR	3.92	3118	1308	332A	THR	1.85	2.68	139.57	3134 [O3]	3084 [O2]
	164A	ARG	3.9	3123	1463	334A	SER	2.62	3	104.47	3102 [O3]	3133[O3]
	331A	PHE	3.59	3112	3073							
	331A	PHE	3.59	3128	3075							

Supplementary Figure S1 (A-E):

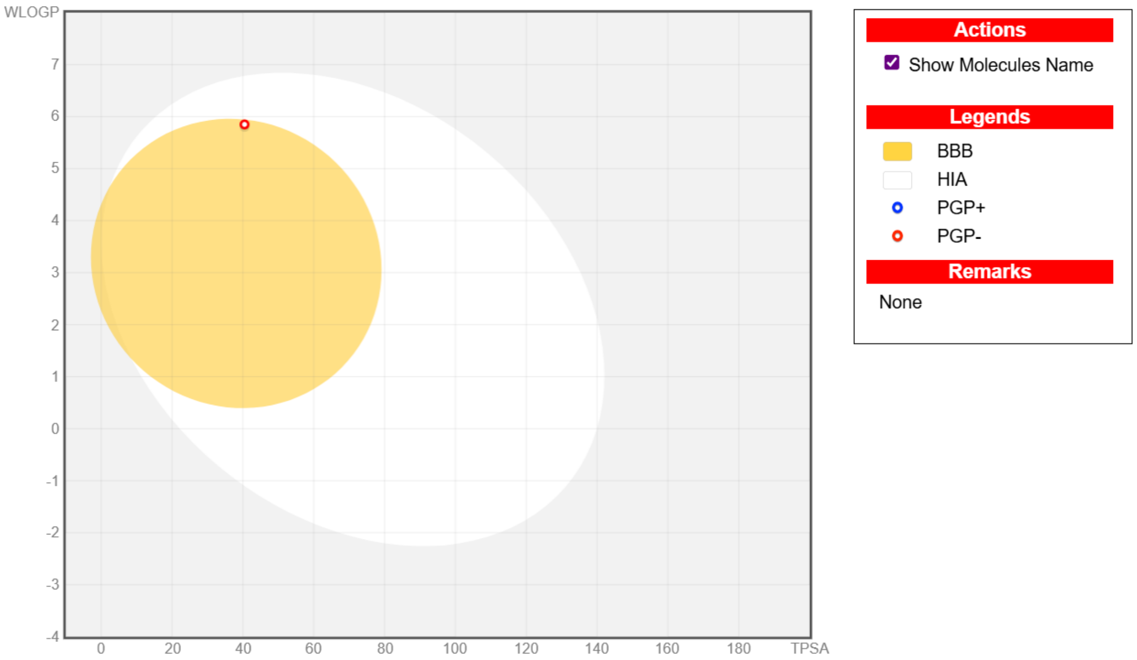

Figure S1A: BOILED-Egg analysis indicates that 1,3-Benzenediol, 2-[3-methyl-6-(1-meth-ylethenyl)-2-cyclohex-en-1-yl]-5-pentyl-, (1R-trans)- exhibits high predicted gastrointestinal absorption, effective blood–brain barrier penetration, and is not a substrate of P-glycoprotein.

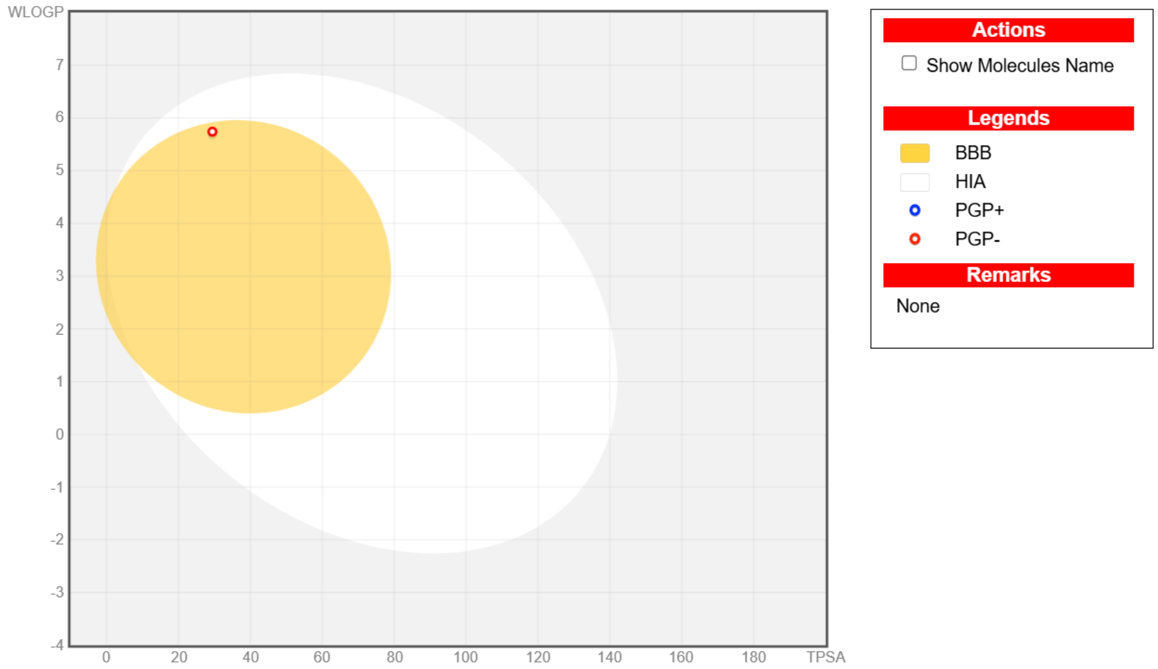

Figure S1B: BOILED-Egg analysis indicates that dronabinol exhibits high predicted gastrointestinal absorption, effective blood–brain barrier penetration, and is not a substrate of P-glycoprotein.

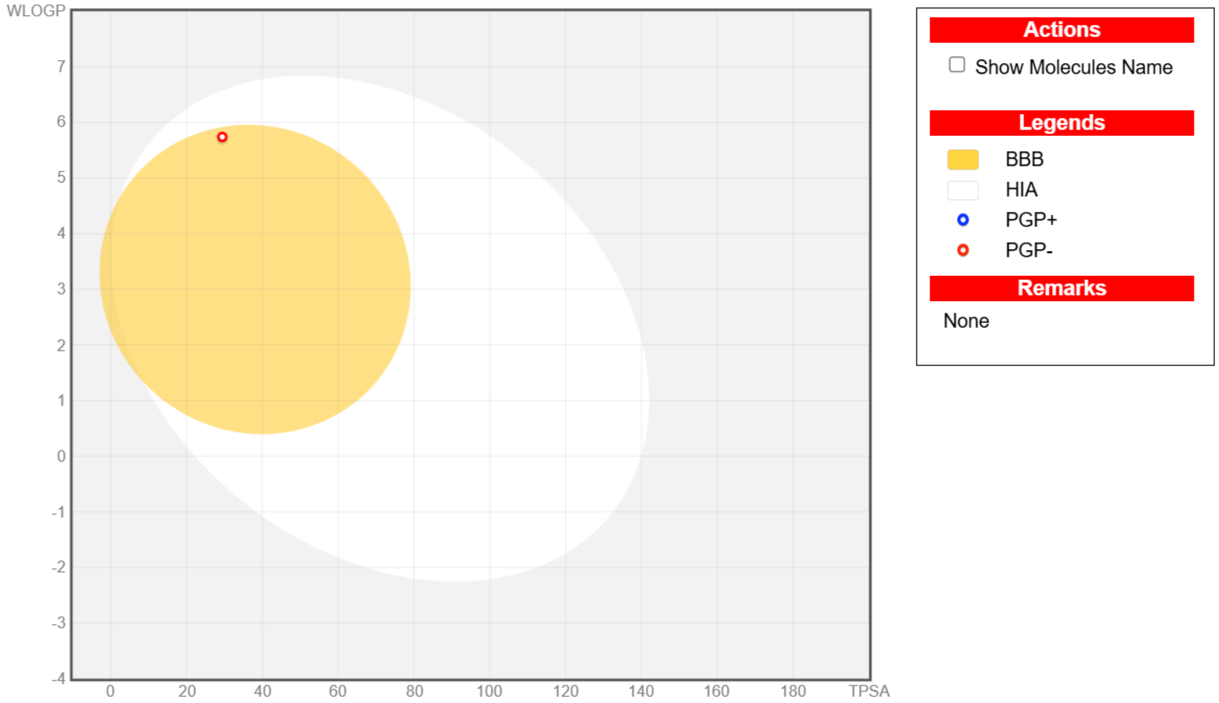

Figure S1C: BOILED-Egg analysis indicates that CBNA exhibits high predicted gastrointestinal absorption, effective blood–brain barrier penetration, and is not a substrate of P-glycoprotein.

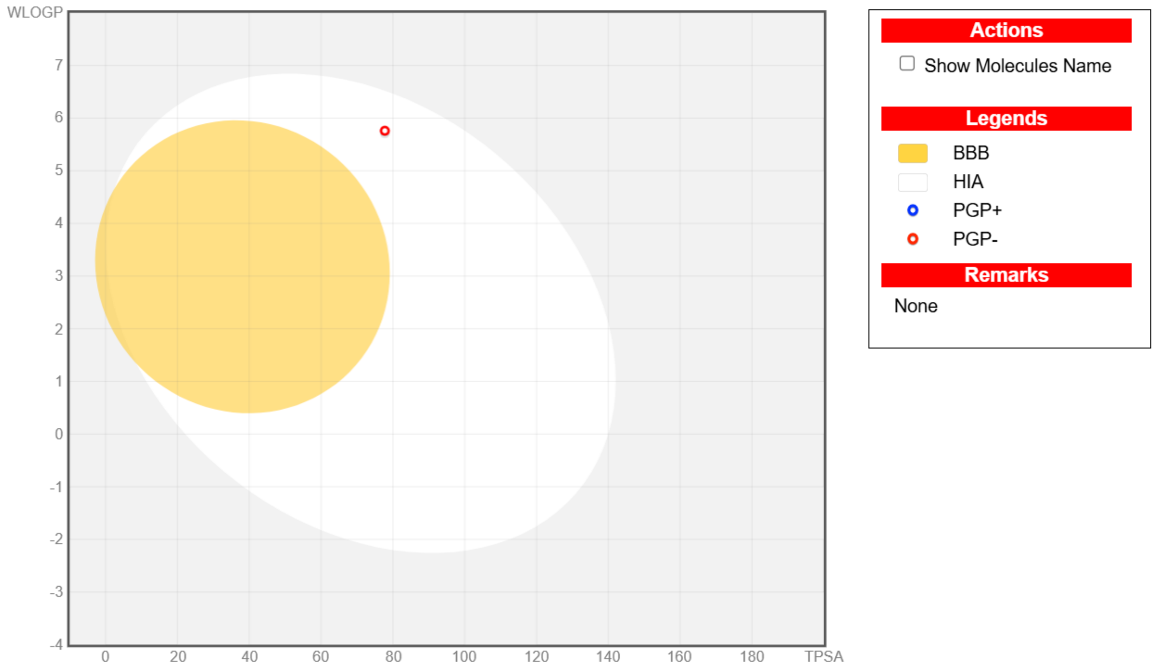

Figure S1D: BOILED-Egg analysis indicates that Cannabigerolic acid (CBGA) exhibits high predicted gastrointestinal absorption, is unlikely to cross the blood–brain barrier, and is not a substrate of P-glycoprotein.

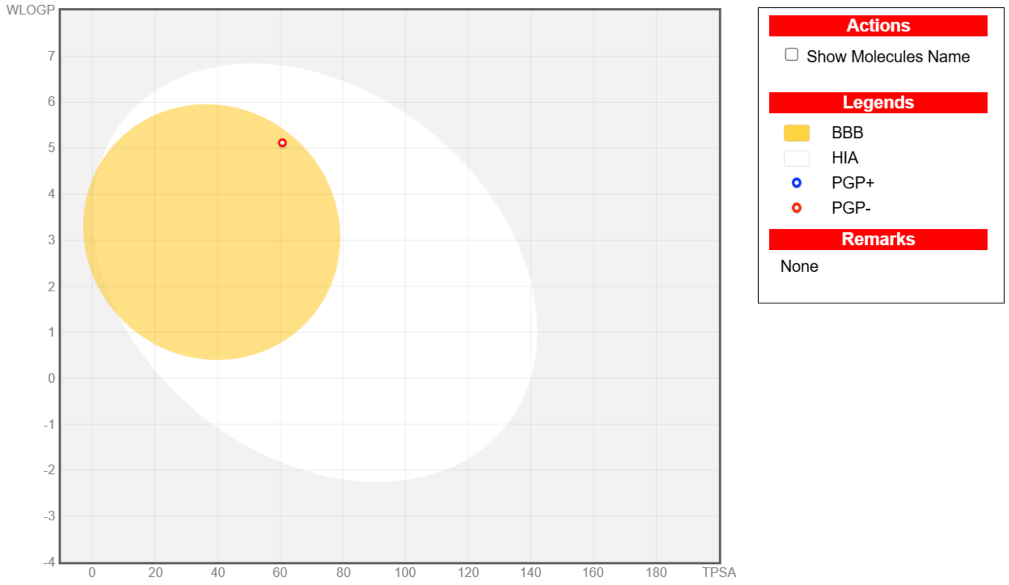

Figure S1E: BOILED-Egg analysis indicates that Ferruginene C exhibits high predicted gastrointestinal absorption, limited blood–brain barrier penetration, and is not a substrate of P-glycoprotein.
